# Cooking by Electricity

**Published:** 1881-06

**Authors:** 


### COOKING BY ELECTRICITY.

Of the many curious things certain to be seen at the forthcoming exhibition of electricity at Paris, not the least remarkable will be the electric cooking range of M. Salignac. That ingenious gentleman is going to fit up his apparatus in the grill room of the restaurant, and intends to furnish a great variety of meats which have been cooked by heat generated from the electric current.

At the last Paris Exhibition, M. Mouchot roasted mutton in condensed sunshine, and literally turned his spit on the hearth of the sun ; but an enthusiastic admirer might say that M. Salignac had far surpassed this in broiling steaks by lightning and warming coffee with the aurora borealis. As a matter of fact, the electric current is as well fitted to produce heat as it is to produce light, and just as electricity will, in all probability be made to yield the principal artificial light of the future, so will doubtless it be applied to household heating. The same machine which light the house by night will heat and cook by day, besides performing other duties, such as driving a coffee mill or a sewing machine.—*Scientific American.*

